# Effects of Hypothalamic Neurodegeneration on Energy Balance

**DOI:** 10.1371/journal.pbio.0030415

**Published:** 2005-11-29

**Authors:** Allison Wanting Xu, Christopher B Kaelin, Gregory J Morton, Kayoko Ogimoto, Kimber Stanhope, James Graham, Denis G Baskin, Peter Havel, Michael W Schwartz, Gregory S Barsh

**Affiliations:** **1**Department of Genetics, Stanford University School of Medicine, Stanford, California, United States of America,; **2**Department of Pediatrics, Stanford University School of Medicine, Stanford, California, United States of America,; **3**Department of Medicine, Harborview Medical Center, University of Washington, Seattle, Washington, United States of America,; **4**Department of Nutrition, University of California, Davis, California, United States of America,; **5**VA Puget Sound Health Care System and University of Washington School of Medicine, Seattle, Washington, United States of America; Beth Israel Deaconess Medical CenterUnited States of America

## Abstract

Normal aging in humans and rodents is accompanied by a progressive increase in adiposity. To investigate the role of hypothalamic neuronal circuits in this process, we used a Cre-lox strategy to create mice with specific and progressive degeneration of hypothalamic neurons that express agouti-related protein (Agrp) or proopiomelanocortin (Pomc), neuropeptides that promote positive or negative energy balance, respectively, through their opposing effects on melanocortin receptor signaling. In previous studies, Pomc mutant mice became obese, but Agrp mutant mice were surprisingly normal, suggesting potential compensation by neuronal circuits or genetic redundancy. Here we find that Pomc-ablation mice develop obesity similar to that described for Pomc knockout mice, but also exhibit defects in compensatory hyperphagia similar to what occurs during normal aging. Agrp-ablation female mice exhibit reduced adiposity with normal compensatory hyperphagia, while animals ablated for both Pomc and Agrp neurons exhibit an additive interaction phenotype. These findings provide new insight into the roles of hypothalamic neurons in energy balance regulation, and provide a model for understanding defects in human energy balance associated with neurodegeneration and aging.

## Abstract 2

Mice are genetically engineered for the progressive degeneration of hypothalamic neurons containing the neuropeptides Pomc and Agrp. Their phenotypes suggest a model for energy balance changes associated with aging.

## Introduction

The mechanisms by which warm-blooded animals balance energy intake with energy expenditure have come under increasing scrutiny in recent years. Important components of this process are the changes associated with aging. In humans, rats, and mice, young adults exhibit a remarkable ability to regulate fuel stores so that the net change in adiposity over long periods of time is a small fraction of energy consumed. As part of the normal aging process, however, a slow but progressive increase in adiposity occurs throughout most of adulthood (reviewed in [1,2]). In both humans and rodents, these changes occur independently of environmental variation, and are likely to be caused by the progressive impairment of mechanisms that normally control energy homeostasis [3–5].

Studies in rodents suggest the underlying causes of age-related changes in energy homeostasis are likely to involve the central melanocortin system [5–7], in which two subgroups of neurons in the arcuate nucleus of the hypothalamus, those marked by the expression of proopiomelanocortin (Pomc) or agouti-related protein (Agrp), sense total body adiposity by sampling circulating indicators such as leptin and insulin. Pomc and Agrp neurons integrate and relay this information to downstream central nervous system effectors that act to balance energy stores via changes in both energy intake and expenditure, with activation of Pomc neurons promoting negative energy balance, and activation of Agrp neurons promoting positive energy balance [8,9].

To some extent, experimental mouse genetic manipulations support this view: deletion of the Pomc gene [10] or transgenic overexpression of Agrp [11] causes hyperphagia and obesity. The results of knockout experiments, however, have been more difficult to interpret. Agrp-deficient mice have been reported to have no detectable defects in energy balance [12], although treatment of adult mice with RNAi against Agrp causes a transient decrease in energy expenditure [13].

An important consideration in interpreting the results of these experiments is the relationship between the action of Pomc or Agrp and that of the Pomc- or Agrp-expressing neurons. The view of energy homeostasis as a system based on neuronal subtypes rather than neuropeptides per se derives in part from the observations that Pomc and Agrp are each coexpressed with other neuropeptides that have similar effects, Cart and neuropeptide Y (Npy), respectively [8], and in part from the observations that the different neuronal subtypes exhibit reciprocal patterns of electrical and/or signaling activity [14,15]. From this perspective, perturbing Pomc or Agrp gene expression may not fully reveal the function of the Pomc or Agrp neuron, respectively.

To better understand the role of Pomc and Agrp neurons in energy balance, and to model how altered activity of these neurons might account for age-related changes in adiposity, we constructed animals in which Pomc- or Agrp-expressing cells were progressively ablated by deleting the mitochondrial transcription factor A *(Tfam)* gene using a Cre-lox strategy. Tfam activity is required for mitochondrial genome transcription and replication, and Tfam-deficient cells exhibit progressive loss of cellular respiration and eventual cell death [16,17]. Here we report that animals in which the *Tfam* gene is removed from Pomc- or Agrp-expressing cells exhibit adult-onset defects in energy balance that provide insight into the normal role of specific hypothalamic neuronal subtypes. Our findings demonstrate that central nervous system regulation of body weight by Agrp neurons involves compensation by redundant genes, and provide a model for understanding defects in energy balance associated with aging and neurodegeneration.

## Results

### Construction and Characterization of Pomc- and Agrp-Neuronal Ablation Animals

In previous experiments using a Cre recombinase transgene controlled by Pomc or Agrp regulatory elements, we demonstrated that expression of the transgene was specific for the different neuronal subtypes in adult animals [9]. To further characterize the timing and pattern of transgene expression, we examined different tissues from mice carrying the PomcCre or AgrpCre transgene together with the lacZ reporter allele *Gt(Rosa)26Sor^tm1Sor^ (R26R)* [18].

In the arcuate nucleus of the hypothalamus, activation of *R26R* by the PomcCre transgene became apparent by embryonic day 17.5; activation of *R26R* by the AgrpCre transgene, however, was not detectable until 2–3 wk of age (Figure 1). These results correspond to what has been described previously for normal development of the melanocortinergic system, with the hypothalamic Pomc system becoming established during fetal brain development, but the Agrp system developing after birth [19,20]. Activation of *R26R* by the PomcCre transgene also occurs in the nucleus tract solitarius of the hindbrain, and the anterior and intermediate lobe of the pituitary in neonatal animals (unpublished data), which are areas in which Pomc is normally expressed. The early onset of Pomc expression in the arcuate nucleus suggests that the Pomc neurons may play a role in the development of the hypothalamus. Expression of Pomc has also been reported in the skin, but we did not detect activation of the *R26R* transgene outside the brain or pituitary gland.

**Figure 1:**
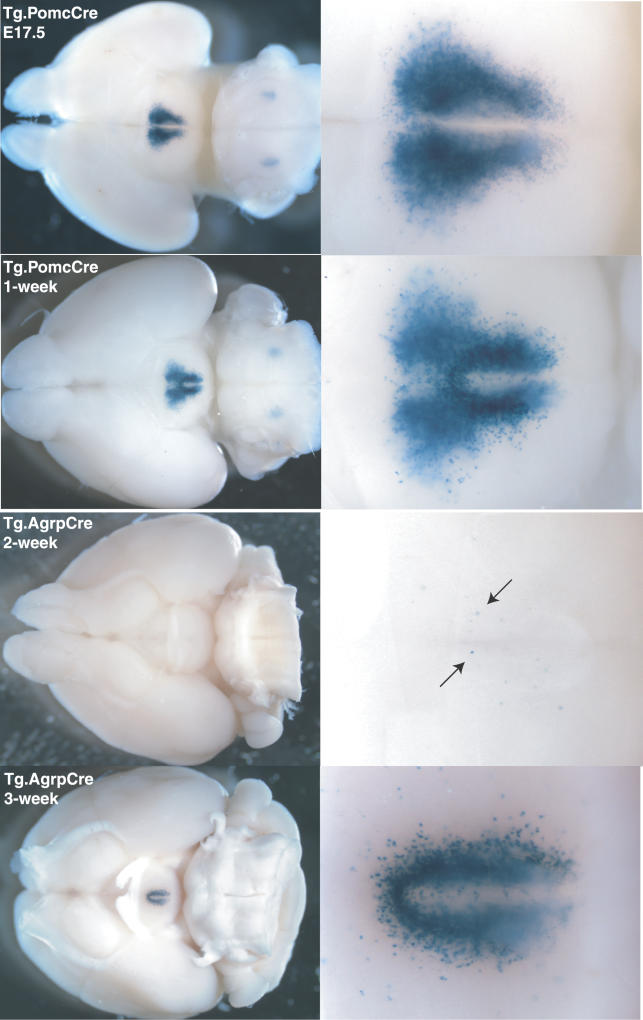
Pattern of *R26R* Activation in Mice Carrying the PomcCre or AgrpCre Transgene Whole brains from *Tg.PomcCre*/+; *R26R*/+ or *Tg.AgrCre*/+; *R26R*/+ mice of the indicated age were fixed and stained with Xgal as described in [9]. Photographs show the ventral brain surface, and coronal sections (not shown) indicate that Xgal-stained neurons lie in the arcuate nucleus of the hypothalamus. As assessed by the extent of Xgal staining, Cre-induced recombination in Pomc neurons is complete by embryonic day 17.5; however, Cre-induced recombination in Agrp neurons is just beginning at 2 wk of age (arrows) and is not complete until 3–4 wk of age.

The loxP-flanked *Tfam* allele, *Tfam^flox^,* was originally constructed by Larsson and colleagues [16] and has been used for a number of cell type–specific ablation studies [17,21,22]. Animals with global *Tfam* deficiency die in early embryogenesis, but animals with *Tfam* deficiency in the neocortex are normal until 5–6 mo of age, when animals die suddenly with massive neuronal cell death. In these latter studies, Cre recombinase was driven by regulatory elements from the calcium/calmodulin-dependent protein kinase II alpha gene *(Camk2a),* which is maximally expressed at 1 mo of age, and the 4- to 5-mo delay between *Tfam* deficiency and neuronal cell death is thought to be due to the gradual depletion of mitochondrial gene products from postmitotic cells [17]. Thus, we anticipated that ablation of Pomc or Agrp neurons due to loss of *Tfam* would be complete by 6 mo of age.

We intercrossed *Tfam ^flox^* ^/*flox*^ and *Tg.PomcCre*/+; *R26R*/+ or *Tg.AgrpCre*/+; *R26R*/+ animals, and identified groups of F2 animals as mutant ablation (*Tfam ^flox^* ^/*flox*^; *Tg.Cre*/?), Tfam control (*Tfam ^flox^*/*^+^*; *Tg.Cre*/?), or Cre recombinase control (*Tfam ^flox^*/*^flox^*; +/+). Consistent with our earlier characterization of the PomcCre and AgrpCre transgenic lines, we observed no phenotypic differences in the different types of control animals; in the studies described below, results for Tfam control and Cre recombinase control animals are pooled. We also generated animals deficient for both Pomc and Agrp cells (*Tfam ^flox^* ^/*flox*^; *Tg.PomcCre*/+; *Tg.AgrpCre*/+), referred to below as “double-ablation” mice.

We examined the pattern and extent of immmunohistochemical staining for α–melanocyte-stimulating hormone (α-MSH) and Agrp in Tfam mutant and control mice at multiple time points. At 2 and 4 mo of age, α-MSH and Agrp neurons in Tfam mutant mice appeared no different from control mice (Figure 2). However, at 7 mo of age, we found that in Pomc-specific Tfam mutant mice, immunohistochemical staining for α-MSH was dramatically reduced compared to control animals, but immunohistochemical staining for Agrp was unaffected (Figure 3). Similarly, in Agrp-specific Tfam mutant mice, immunohistochemical staining for Agrp was dramatically reduced compared to control animals, but immunohistochemical staining for α-MSH was unaffected (Figure 3). In both cases, there was no detectable gliosis or effect on brain architecture, indicating that the ablation was highly specific.

**Figure 2:**
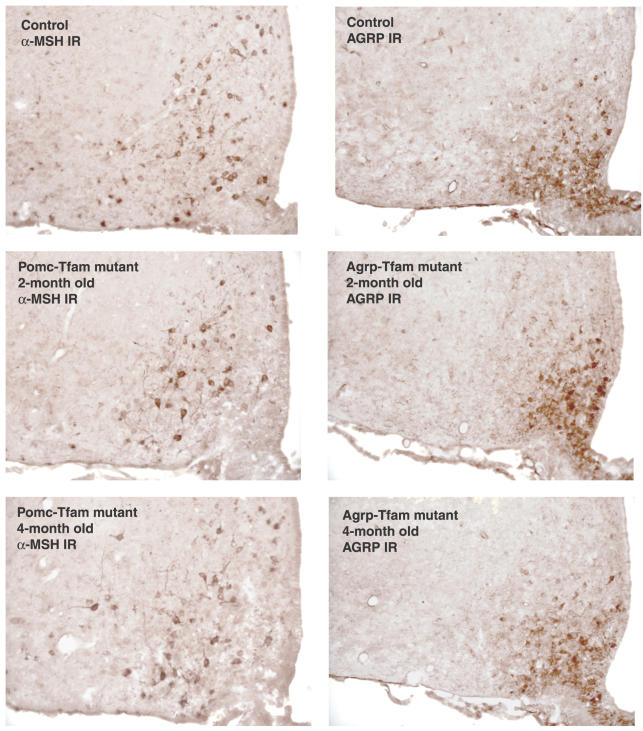
Immunostaining of α-MSH or Agrp in Control and Tfam Mutant Animals of Different Ages Hypothalami from control (*Tfam ^flox^*/*^+^*; *Tg.PomcCre*/+ or *Tfam ^flox^*/*^flox^*; +/+), Pomc-specific Tfam mutant (*Tfam ^flox^*/*^flox^*; *Tg.PomcCre*/+), and Agrp-specific Tfam mutant (*Tfam ^flox^*/*^flox^*; *Tg.AgrpCre*/+) animals were harvested at the indicated ages and immunostained for α-MSH or Agrp as indicated. IR, immmunoreactivity.

**Figure 3:**
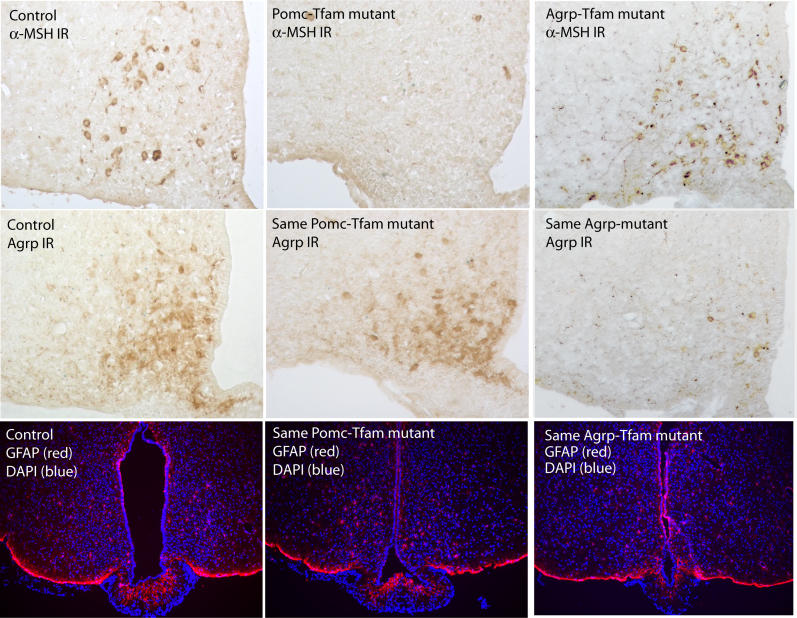
Effect of Pomc-Specific Tfam Deficiency on Pomc and Agrp Immunostaining Each column of panels shows coronal sections from a 7-mo-old control (*Tfam ^flox^*/*^+^*; *Tg.PomcCre*/+ or *Tfam ^flox^*/*^flox^*; +/+), Pomc-specific Tfam mutant (*Tfam ^flox^*/*^flox^*; *Tg.PomcCre*/+), and Agrp-specific Tfam mutant (*Tfam ^flox^*/*^flox^*; *Tg.AgrpCre*/+) on the left, middle, and right, respectively. Each section was stained for α-MSH or Agrp as indicated. The three lower panels show coronal sections stained to reveal cell nuclei (DAPI) and immunostained for GFAP, and indicate that ablation of Pomc or Agrp neurons does not grossly disturb tissue architecture, cell number, or stimulate astrocytosis. The sections shown are representative of three different animals that were examined. IR, immmunoreactivity.

As an additional measure of ablation, we examined Xgal staining of hypothalamic sections from experimental and control mice that carried the *R26R* reporter gene, since, for example, loss of Agrp neurons from *Tg.AgrpCre*/+; *Tfam ^flox^* ^/*flox*^ ; *R26R*/+ mice should be reflected in a reduced number of Xgal-positive neurons. For both Pomc-specific and Agrp-specific Tfam mutant mice, very few Xgal-positive cells were detectable; furthermore, mRNA in situ hybridization revealed a dramatic reduction in the number of *Agrp*-expressing cells (Figure 4). Thus, loss of Tfam induced by a PomcCre or AgrpCre transgene gives rise to postnatal loss of Pomc or Agrp neurons that is specific and extensive.

**Figure 4:**
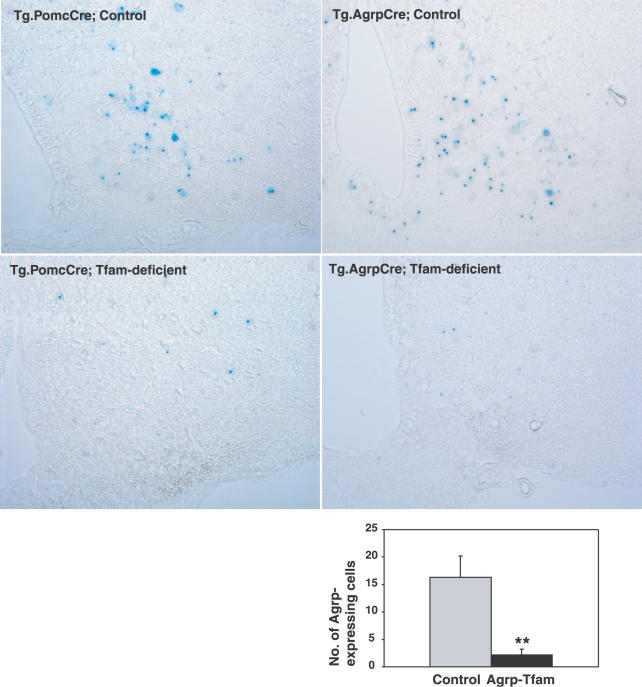
Expression of the R26R Cre Reporter Gene in Pomc- or Agrp-Specific Tfam Mutant Animals Animals carrying the PomcCre or AgrpCre transgene together with the lacZ reporter allele for Cre recombination *Gt(Rosa)26Sor^tm1Sor^* (R26R) were intercrossed with *Tfam ^flox^*/*^flox^* animals. F2 progeny of the indicated genotype were sacrificed at 7 mo of age, and hypothalamic coronal sections stained for Xgal. In this situation, Xgal staining serves as an autonomous histologic marker, and loss of Xgal staining in Tfam mutant animals is therefore secondary to cell death. Sections are representative of three to four per genotype group that were examined. The panel on the lower right shows the number of Agrp-expressing neurons in the control and Agrp-Tfam mutants as determined by fluorescence in situ hybridization to *Agrp* mRNA. **, *p* ≤ 0.01. Error bars = standard error of the mean.

### Effects of Pomc and/or Agrp Ablation on Energy Balance and Neuroendocrine Axis

Pomc-ablation mice exhibited a progressive adult-onset obesity in which increased weight gain compared to controls was first detectable at 4 mo of age, and the magnitude of the effect was greater in females (∼30% increase in body weight) than in males (∼15% increase in body weight) (Figure 5A and 5B). Agrp-ablation mice exhibited little difference in body weight compared to control animals, although at every time point, the mean body weight for mutant animals was slightly less than control animals for both males (Figure 5C) and females (Figure 5D). A more significant role for Agrp neurons in total body weight was uncovered by examining double-ablation mice; animals deficient for both neuronal subtypes weigh more than control animals but less than Pomc single-ablation mice in both males (Figure 5E) and females (Figure 5F).

**Figure 5:**
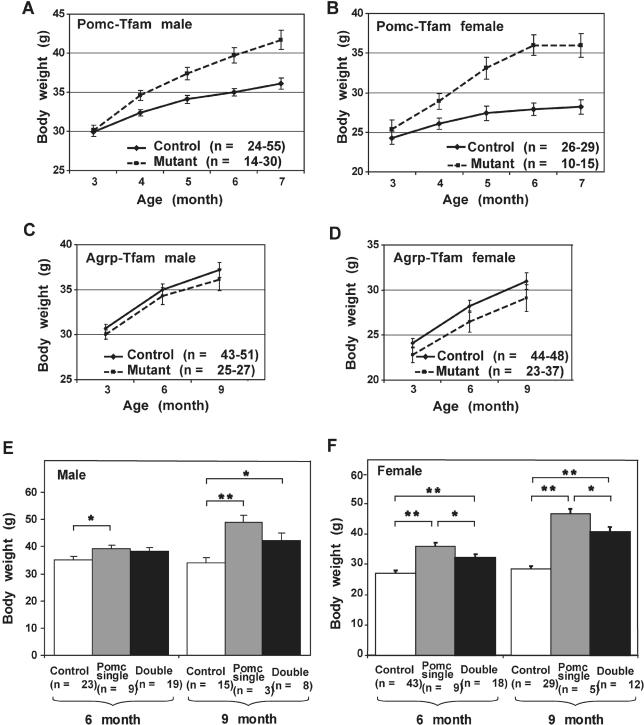
Effect of Pomc- and/or Agrp-Specific Tfam Mutations on Body Weight (A–D) show longitudinal measurements of body weight in animals of the indicated genotype and sex. (E) and (F) show comparisons of single (Pomc-specific) and double (Pomc- and Agrp-specific) Tfam deficiency for 6- and 9-mo-old animals. *, *p* ≤ 0.05; **, *p* ≤ 0.01. Error bars = standard error of the mean.

Using dual energy X-ray absorptiometry (DEXA) or MRI to measure adipose versus nonadipose tissue, we observed that in 7- to 10-mo-old animals, loss of Pomc-expressing cells caused an increase in both compartments, with a relative effect that was slightly greater on fat than on lean body mass (Figure 6A and 6B). Agrp ablation caused a small but significant decrease of fat mass in females but not in males (Figure 6D). We also measured rates of food intake and energy expenditure in Pomc-ablation animals, and observed an effect on both components of the energy balance equation. Pomc-ablation mice consumed approximately 10% more than control animals, and exhibited rates of O_2_ consumption approximately 10% less than control animals (Figure 7).

**Figure 6:**
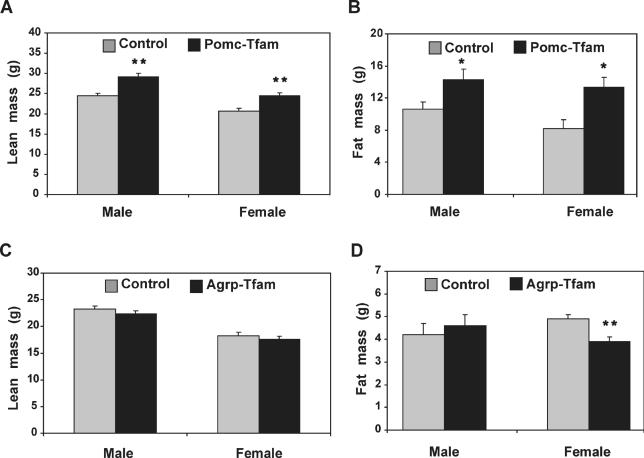
Lean Body Composition of Animals with Pomc- or Agrp-Specific Tfam Deficiency Lean body mass of animals of the indicated genotype was determined as described in Materials and Methods. For Pomc-specific Tfam mutant animals (A), numbers of animals were: control *n* = 10, mutant *n* = 4 (male); and control *n* = 6, mutant *n* = 4 (female). For Agrp-specific Tfam mutant animals (C), numbers of animals were control *n* = 4, mutant *n* = 4 (male); and control *n* = 6, mutant *n* = 8 (female). (B) and (D) show comparisons of control and mutant animals of the indicated genotype and sex for fat masses of 7- to 10-mo-old animals. For Pomc-specific Tfam mutant animals (B), numbers of animals were control *n* = 10, mutant *n* = 4 (male); and control *n* = 6, mutant *n* = 4 (female). For Agrp-specific Tfam mutant animals (D), numbers of animals were control *n* = 4, mutant *n* = 4 (male); and control *n* = 6, mutant *n* = 8 (female). *, *p* ≤ 0.05; **, *p* ≤ 0.01. Error bars = standard error of the mean.

**Figure 7:**
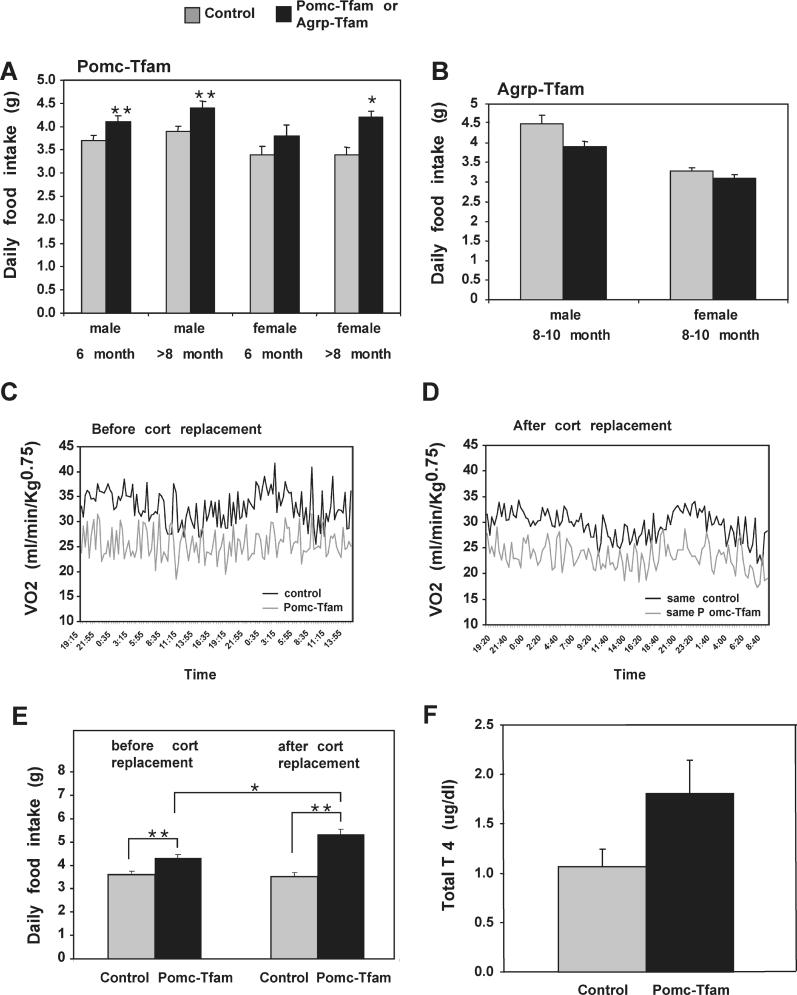
Food Intake and Energy Expenditure in Animals with Pomc- or Agrp-Specific Tfam Deficiency (A and B) Daily food intake was measured for 7 d as described in Materials and Methods. For (A), numbers of animals used (control and Pomc-Tfam mutant) were 6-mo-old male (*n* = 13, *n* = 7); 6-mo-old female (*n* = 10, *n* = 6); > 8-mo-old male (*n* = 10, *n* = 6); and > 8-mo-old female (*n*= 13, *n* = 6). (C, D) O_2_ consumption was measured over a 24-h period as described in Materials and Methods; panels illustrate results from a single control and a single Pomc-Tfam mutant animal before and after (10–12 d) corticosterone replacement; these results are representative of four control and four mutant animals that were examined. (E) Daily food intake was significantly increased after corticosterone replacement in Pomc-Tfam mutant animals (*n* = 6) but not in control (*n* = 9) animals. (F) Measurement of total serum T4 levels in control (*n* = 7) and Pomc-Tfam mutant (*n* = 5) animals. *, *p* ≤ 0.05; **, *p* ≤ 0.01. Error bars = standard error of the mean.

The Pomc-ablation mice lost Pomc-expressing pituitary cells in addition to Pomc neurons, and therefore had chronic ACTH insufficiency with reduced basal corticosterone levels (unpublished data). Although both Pomc knockout and the Pomc-ablation mice showed corticosterone deficiency due to lack of ACTH, the Pomc-ablation mice developed adrenal glands (unpublished data), whereas adrenal glands in the Pomc knockout mice were largely absent [10]. We used long-term corticosterone replacement to investigate whether or not hypoadrenalism contributed to abnormal energy balance in Pomc-ablation mice. Corticosterone pellets (10 mg; 21 d) were implanted into both Pomc-ablation and control mice, and measurements of food intake and energy expenditure were made after 10–12 d. In control animals, corticosterone replacement had no effect on energy expenditure (Figure 7C and 7D) or food intake (Figure 7E). In Pomc-ablation animals, corticosterone replacement had no effect on energy expenditure (Figure 7C and 7D) but stimulated food intake by approximately 20% over a level that was already elevated relative to control (Figure 7E). These observations suggest that ablation of Pomc neurons is sufficient to reduce energy expenditure and increase food intake; in fact, concomitant adrenocortical deficiency in Pomc-ablation mice would, if anything, mitigate the obesity phenotype.

α-MSH is also thought to stimulate the thyroid axis via thyroid-releasing hormone, but measurements of total T4 in Pomc-ablation mice were not significantly different from those of control mice (Figure 7F).

### Impaired Compensatory Refeeding in Pomc-Ablation Mice

Because Pomc neurons serve as primary hypothalamic sensors for adiposity signals, we suspected that hyperphagia and reduced energy expenditure in Pomc-ablation mice were caused by an underlying abnormality in the hypothalamic circuitry that normally maintains peripheral energy stores. To test this idea directly, we examined the ability of 6-mo-old mutant and control animals to increase food intake acutely following a period of food deprivation. After a 48-h fast, animals normally compensate with a transient hyperphagia sufficient to restore adipose depots to prefasting levels, a phenomenon typically associated with a 2-fold increase in food intake over 24 h. We found that Pomc-ablation mice exhibit an impaired compensatory refeeding response, consuming approximately 25% less than expected (Figure 8A); conversely, Agrp-ablation mice exhibited a normal refeeding response (Figure 8B). These effects also were apparent in weight gain after refeeding; control and Agrp-ablation mice were able to recover their body weight precisely within 24 h after fasting, while Pomc-ablation mice were not (Figure 8C and 8D).

**Figure 8:**
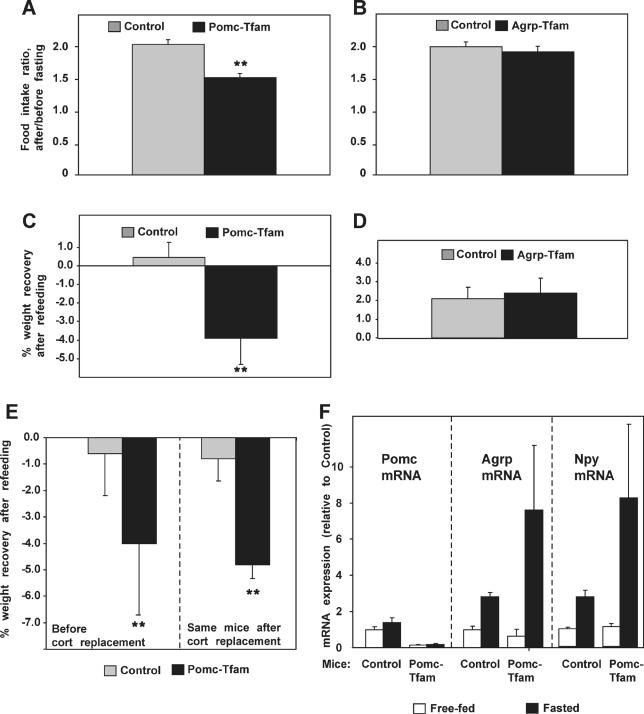
Compensatory Refeeding and Neuropeptide mRNA levels in Pomc-Specific and Agrp-Specific Tfam Deficiency (A–D) 24-h compensatory refeeding after a 48-h fast was measured as described in Materials and Methods; data are shown either as (A and B) the ratio of food consumed over 24 h (refeeding) to normal daily food intake (averaged over 7 h prior to food deprivation), or as (C and D) percentage of weight recovery after 24 h of refeeding. For the Pomc-Tfam experiment (A and C), number of animals used was *n* = 23 (control) and *n* = 13 (Pomc-Tfam mutant); for the Agrp-Tfam experiment (B and D), number of animals used was *n* = 21 (control) and *n* = 15 (Agrp-Tfam). (E) The same refeeding defects were observed in Pomc-Tfam mutants after corticosterone replacement (control, *n* = 9; Pomc-Tfam mutant, *n* = 6). (F) Expression of Pomc, Agrp, and Npy in Pomc-Tfam mutants. Mice were fasted for 48 h 10 d after implanting corticosterone pellets, and expression of Pomc, Agrp, and Npy in the hypothalamus was measured by semi-quantitative RT-PCR. Values are shown as relative levels compared to free-fed controls. Numbers of animals used were *n* = 5 (control fed); *n* = 5 (control fasted); *n* = 3 (mutant fed); and *n* = 3 (mutant fasted). *, *p* ≤ 0.05; **, *p* ≤ 0.01. Error bars = standard error of the mean.

To investigate the extent to which adrenocortical impairment might contribute to the refeeding abnormality in Pomc-ablation mice, we carried out the experiment before and after implanting corticosterone replacement pellets. Although this approach cannot completely mimic the normal circadian and metabolic fluctuations of glucocorticoids, we found that during the period of corticosterone replacement, blood corticosterone levels were increased after fasting in both controls and mutants (unpublished data), indicating that the adrenal glands of the Pomc-ablation mice were able to mount a stress response. Even though corticosterone replacement stimulated food intake (Figure 7E) and weight gain (unpublished data), mutant mice were (Figure 8E) unable to compensate for body weight lost by fasting.

*Agrp* and *Npy* mRNA levels are normally upregulated by fasting, and have been suggested to contribute to compensatory refeeding. Surprisingly, we found that Pomc-ablation mice exhibited a normal response to fasting in terms of their ability to upregulate *Agrp* and *Npy* mRNA (Figure 8F). These findings suggest that impaired compensatory refeeding in Pomc-ablation mice is caused not by inability to upregulate orexigenic neuropeptides, but instead by the absence of Pomc neurons. Thus, Pomc-ablation mice represent an interesting paradox in which mutant animals are hyperphagic at baseline, yet are unable to increase their food intake to compensate for food deprivation.

## Discussion

Although physical or chemical lesioning studies carried out 60 years ago first drew attention to the ventromedial area and the lateral hypothalamic area of the hypothalamus as sites important for regulating energy balance [23,24], functional heterogeneity within the arcuate nucleus of the hypothalamus was not recognized until recently. Our results address functional heterogeneity in a specific way by ablating subpopulations of neurons with a Cre-lox strategy. Distinguishing between the action of a neuropeptide and the action of a neuropeptide-specific cell is also apparent by considering previous studies of *Agrp* and *Pomc* knockout mice. In contrast to *Agrp* knockout or *Agrp*; *Npy* double-knockout mice, which are normal [12], Agrp-ablation mice show reduced adiposity. This reduction in adiposity is most apparent in the Agrp; Pomc double-ablation mice, an observation that is reminiscent of what was previously described for the interaction of *Npy* and *Lep^ob^* [25]. This observation points to an important role for other neuropeptides and neurotransmitters besides Agrp and Npy. One such candidate is gamma-aminobutyric acid, which has been shown to be released from Npy neurons and inhibit the activity of Pomc neurons [14]. The relatively mild weight phenotype of the *Agrp*-ablation mice could be due to a small fraction of the Agrp neurons that escape ablation, differences in genetic background, potential compensation developed during the course of progressive cell loss, or a combination of these mechanisms. One such potential compensatory mechanism is neuronal plasticity and rewiring of the feeding circuits as described by Horvath and colleagues [26].

Nonetheless, our findings and similar results recently published by Bewick et al. [27], Gropp et al. [28], and Luquet et al. [29] demonstrate that ablation of Agrp neurons yields a phenotype that is more dramatic than a knockout of Agrp (or combined ablation of Agrp and Npy [12]). Furthermore, acute ablation of the Agrp neurons over a time frame of days [28,29] yields a more severe phenotype than progressive ablation of the Agrp neurons over a time frame of months due to neurodegeneration conferred by Tfam deficiency. These observations highlight the potential for neuronal plasticity and developmental compensation [26], and have implications for both experimental strategies and therapeutic approaches to energy homeostasis. By contrast, *Pomc* knockout mice [10,30,31] exhibit a phenotype that is more dramatic than ablation of Pomc neurons, with obesity starting at 4–8 wk of age and an eventual ∼50% increase in body weight, whereas Pomc-ablation mice develop obesity starting at 4 mo of age with an eventual 15%–30% increase in body weight (Figure 5). The later time of onset can be attributed simply to the difference between germline inheritance of a knockout allele and somatic loss of mitochondrial function in specific cells, but several factors could account for the difference in magnitude, including an inbred versus a mixed genetic background, a small fraction of Pomc neurons that escape ablation, or additional products released by Pomc neurons that counter the effects of Pomc.

The surprising result that the Pomc-ablation mice failed to defend their body weight normally after food deprivation is somewhat paradoxical given their chronic hyperphagia and obesity. We propose that normal compensatory refeeding requires either downregulation of Pomc neuronal activity, decreased Pomc release, or both, and that normal downregulation is abolished in Pomc-ablation mice. This suggestion is consistent with the observation that widespread transgenic overexpression of *Pomc* also gives rise to impaired compensatory refeeding thought to be due to disruptions of Pomc regulation; however, these animals exhibit a chronic state of negative rather than positive energy balance [32].

Electrophysiologic studies have suggested a melanocortinergic neuronal circuit in which Agrp neurons control the activity of Pomc neurons [14,33], but the reciprocal question—do Pomc neurons control the activity of Agrp neurons—has not been addressed. However, Agrp and Npy mRNA levels responded normally to food deprivation in our Pomc-ablation mice, which indicates that a potential pathway from Pomc neurons to Agrp neurons, should one exist, does not act by controlling production of Agrp or Npy.

Our strategy for genetic ablation of Pomc and Agrp neurons is based on a prior study in which Cre-mediated loss of *Tfam* driven by the *Camk2a* promoter caused widespread neuronal cell death due to loss of mitochondrial ATP production [17]. In some ways, this approach is similar to that used by Sakurai and colleagues [34] for the ablation of hypocretin/orexin (Hcrt)-expressing neurons, in which transgenic expression of a polyglutamine-containing protein caused *Hcrt*-expressing neurons to die in the immediate postnatal period. In fact, while this manuscript was under review, Bewick et al. [27] reported that transgenic animals carrying a polyglutamine-containing protein driven by the *Agrp* promoter exhibit reduced adiposity and body weight, similar to our observations. The later onset of neuronal cell death induced by *Tfam* deficiency is likely explained by a gradual depletion of mitochondrial gene products from postmitotic cells, and therefore provides a model for pathophysiologic changes related to the mitochondrial theory of aging, in which progressive impairment of mitochondrial function associated with oxidative damage is thought to lead to a vicious circle that culminates in neuronal apoptosis [35–37]. In a recent test of this model, Trifunovic et al. [38] found that animals genetically engineered to accumulate mitochondrial mutations developed several features of premature aging. While not specifically tested by Trifunovic et al. [38], our results predict that these animals should exhibit a defective energy balance with an impairment in compensatory hyperphagia.

From this perspective, the defective energy balance that developed in our Pomc-ablation or double-ablation mice at 4 mo of age provides a useful model for age-related obesity. In both rodents and humans, adiposity progressively increases throughout most of adult life. Even in elderly individuals, where total body weight (and subcutaneous fat) declines toward the end of life, visceral adiposity continues to increase, pointing to a fundamental age-related defect in energy balance [1,3,5]. Elderly humans exhibit defects in both the normal reduction in energy expenditure caused by overfeeding [39], and the normal increase in perceived hunger caused by underfeeding [40]. Defects in compensatory hyperphagia similar to what we observed in our Pomc-ablation or double-ablation mice have also been described in studies of young (3-mo-old), middle-aged (12-mo-old), and elderly (24-mo-old) rats from Matsumoto and colleagues [6,41]. Notably, impaired energy homeostasis in the rat model is associated with altered expression of Pomc [6] and Npy [41]. Thus, the neuronal-ablation mice we describe here represent a model for premature dysfunction of hypothalamic circuits implicated in the normal aging process.

## Materials and Methods

### Animal studies

Animals carrying the *Tfam^loxP^* allele [16,17] were kindly provided by N. Larsson (Karolinska Institute, Stockholm, Sweden) on a mixed C57BL/6J and 129 background. Animals carrying the *R26R* LacZ reporter allele were obtained from P. Soriano (University of Washington, Seattle, Washington, United States), and are maintained in our laboratory as homozygotes on a mixed background that includes contributions from FVB/N, C57BL/6J, and 129 strains. The *Tg.PomcCre* and *Tg.AgrpCre* lines are maintained as heterozygotes on the same mixed background; their construction and characterization has been described previously [9]. We generated a total of 128 animals for the Pomc-ablation mice (44 mutants, 62 Tfam controls, and 22 Cre recombinase controls), 133 animals for the Agrp-ablation mice (44 mutants, 54 Tfam controls, and 35 Cre recombinase controls), and 42 double-ablation mice. To help control for differences in litter size and modifier genes, results were analyzed by two-way ANOVA in which sibship was included as a variable. Although the genetic background was heterogeneous, there was less variation (both environmental and genetic) within litters than between litters.

For feeding studies, animals were singly housed for 2 wk, then food intake was measured for a consecutive 6–7 d; results are shown as an average over 24 h. To measure compensatory refeeding, food was removed 3 h before the onset of the dark cycle, mice were fasted for 48 h, then food was added back and food intake measured for the next 24 h.

Metabolic monitoring was carried out with a system (AccuScan Instruments, Columbus, Ohio, United States) located in the UC Davis Department of Nutrition animal facility. The system consists of an O_2_ analyzer, CO_2_ analyzer, and PhysioScan analyzer, which monitors vertical and horizontal movement via light beam interruption. A flow controller/channelyzer allows for flow rate adjustments and sequential channeling of airflow from a reference line and the four animal chambers through the CO_2_ and O_2_ analyzers. The flow rate for these experiments was 0.5 l/min. The dimensions of the plexiglas chambers were 12 in × 8 in × 8 in. The Integra ME software includes O_2_ and CO_2_ analyzer calibration, data collection, and analysis programs. Reported and calculated values include O_2_ consumption, CO_2_ production, RQ, heat production (energy expenditure), total ambulatory movement, and total rest time. Animals were acclimated to the chambers for 20 h immediately prior to a 24-h data collection period. Each animal received its normal diet and water while in the chamber.

Body fat mass and lean mass were determined either by dual X-ray absorptiometry (Pomc-ablation mice) or by MRI (Agrp-ablation mice). In the former case, mice were anesthetized by intraperitoneal injection of 2.5% avertin, and body composition was measured using a PIXImus2 instrument. In the latter case, conscious animals were analyzed with an MRI whole-body composition analyzer (Echo Medical Systems, Houston, Texas, United States) at the Rodent Energy Metabolism core at the University of Washington.

For studies involving corticosterone replacement, a 10-mg 21-d release corticosterone pellet (Innovative Research of America, Sarasota, Florida, United States) was implanted subcutaneously on the lateral side of the neck of both ablation and control animals. This dose of corticosterone is sufficient for physiologic replacement and has been shown to prevent adrenalectomy-induced anorexia [42].

### Molecular biology

Measurements of mRNA levels were carried out by quantitative RT-PCR on RNA extracted from dissected hypothalamic tissue. Total RNA for each hypothalamus was quantified by spectrophotometry after purification using TRIzol reagent (Invitrogen, Carlsbad, California, United States) and an RNeasy mini kit (Qiagen, Valencia, California, United States). 250 ng each total RNA sample was reverse-transcribed, then PCR-amplified using a Lightcycler (Roche Applied Science, Basel, Switzerland) and SYBR green to measure relative cDNA levels. *Agrp* primers were TGCTACTGCCGCTTCTTCAA and CTTTGCCCAAACAACATCCA; *Npy* primers were TAACAAGCGAATGGGGCTGT and ATCTGGCCATGTCCTCTGCT; *Pomc* primers were AGGCCTGACACGTGGAAGAT and AGGCACCAGCTCCACACAT; and *Actb* primers were CTGCGTTTTACACCCTTTCTTTG and gccatgccaatgttgtctcttat. Efficiency for each primer set was estimated from standard curves made with serial cDNA dilutions.

Xgal staining and immunohistochemistry for α-MSH or AGRP was carried out as described previously [9]. For glial fibrillary acidic protein (GFAP) immunofluorescence, a polyclonal anti-GFAP antibody (Dako, Glostrup, Denmark) was used at 1:1500 dilution according to the protocol described in [9]. Goat–anti-rabbit Alexa488 (Molecular Probes, Eugene, Oregon, United States; 1:200) was used for secondary antibody detection. Sections were mounted using Vectashield with DAPI (Vector Laboratories, Burlingame, California, United States). Fluorescence images were captured using a black-and-white digital camera (AxioCam MRm) attached to a Zeiss Axioplan2 imaging system (Zeiss, Oberkochen, Germany), and later pseudocolored to red (GFAP) and blue (DAPI) and superimposed.

For fluorescence in situ hybridization to *Agrp* mRNA, brains were frozen on dry ice, and 14-μm cryostat sections mounted on slides using RNAse-free conditions. Labeling and hybridization were carried out as described previously [43].

## Abbreviations

α-MSH: α–melanocyte-stimulating hormone
Agrp: agouti-related protein
GFAP: glial fibrillary acidic protein
Npy: neuropeptide Y
Pomc: proopiomelanocortin
